# Prognostic value of CLIC3 mRNA overexpression in bladder cancer

**DOI:** 10.7717/peerj.8348

**Published:** 2020-01-06

**Authors:** Mei Chen, Shufang Zhang, Xiaohong Wen, Hui Cao, Yuanhui Gao

**Affiliations:** Central Laboratory, Affiliated Haikou Hospital of Xiangya Medical College, Central South University, Haikou, Hainan, China

**Keywords:** CLIC3, Bladder cancer, Expression, Prognosis, GSEA

## Abstract

**Background:**

Human intracellular chloride channel 3 (CLIC3) is involved in the development of various cancers, but the expression and prognostic value of CLIC3 mRNA in bladder cancer (BC) remain unclear.

**Methods:**

The gene expression data and clinical information of CLIC3 were obtained from the Gene Expression Omnibus (GEO) database and verified in the Oncomine and The Cancer Genome Atlas (TCGA) database. The expression of CLIC3 mRNA in BC tissues and adjacent normal tissues was detected by quantitative real-time polymerase chain reaction (qRT-PCR). The Kaplan-Meier method was used to analyze the relationship between the expression of CLIC3 mRNA and the prognosis of BC. Cox univariate and multivariate analyses were performed on the overall survival and tumor-specific survival of BC patients. The genes coexpressed with CLIC3 were analyzed by Gene Ontology (GO) and Kyoto Encyclopedia of Genes and Genomes (KEGG). CLIC3-related signal transduction pathways in BC were explored with gene set enrichment analysis (GSEA).

**Results:**

The expression of CLIC3 mRNA in BC tissues was higher than that in normal tissues (*P* < 0.01). High CLIC3 mRNA expression was associated with age (*P* = 0.021) and grade (*P* = 0.045) in BC patients. High CLIC3 mRNA expression predicted a poor prognosis in BC patients (*P* < 0.05). Cox univariate and multivariate analyses showed that high CLIC3 mRNA expression was associated with tumor-specific survival in BC patients (*P* < 0.05). Functional enrichment analyses indicated that CLIC3 may be significantly associated with the cell cycle, focal adhesion, the extracellular matrix (ECM) receptor interaction and the P53 signaling pathway.

**Conclusions:**

CLIC3 mRNA is highly expressed in BC, and its high expression is related to the adverse clinicopathological factors and prognosis of BC patients. CLIC3 can be used as a biomarker for the prognosis of BC patients.

## Introduction

Bladder cancer (BC) is one of the most common malignant tumors in the urogenital system. BC can be divided into muscle invasive BC (MIBC) and non-muscle invasive BC (Kim, 2015). The incidence of BC is increasing, ranking third in male cancer incidence (Siegel, Miller & Jemal, 2017). The high recurrence rate and drug resistance of BC after operation are difficult points in clinical research. Biomarkers are the hotspot in the screening and diagnosis of BC (Kiyoshima et al., 2016). Therefore, finding key genes related to the prognosis of BC is of great significance for monitoring the postoperative recurrence of BC and the addition of new therapeutic targets.

The human chloride intracellular channel (CLIC) family consists of six members, CLIC1 through CLIC6 (Suh & Yuspa, 2005). CLICs proteins mostly participate in macrophage activation (Fernandez-Salas et al., 1999), regulation of intraveliscular pH (Ulmasov et al., 2009), and apoptosis (Xu et al., 2013). Chloride intracellular channel 3 (CLIC3) is a type of intracellular chloride channel that is located at 9q34.3 and consists of 236 amino acids. The encoded protein size is 26.6 ku, which can promote chloride ion motion (Money et al., 2007; Qian et al., 1999).

CLIC3 is distributed in a variety of tissues, such as the placenta, lung and heart, and exists in a small amount in skeletal muscle (Murthi et al., 2012). Previous studies have found that CLIC3 is closely related to the apoptosis and migration of tumor cells and plays an important role in the occurrence and development of tumors (Kunzelmann, 2005; Dozynkiewicz et al., 2012). CLIC3 can drive cancer progression through glutathione-dependent oxidoreductase activity (Hernandez-Fernaud et al., 2017). CLIC3 is highly expressed in a variety of tumors, such as mucoepidermoid carcinoma (Wang et al., 2015) and malignant pleural mesothelioma (Tasiopoulou et al., 2015). However, the expression of CLIC3 in BC has not yet been reported.

In this study, the expression of CLIC3 mRNA and its clinical characteristics and prognosis in patients with BC were analyzed using data from databases. CLIC3-related signal transduction pathways in BC were analyzed by gene set enrichment analysis (GSEA), which provided clues and ideas for further study of the mechanism of CLIC3 in the occurrence and development of BC. Finally, quantitative real-time polymerase chain reaction (qRT-PCR) results further confirmed that CLIC3 mRNA expression in BC was higher than that in normal tissues.

## Material and Methods

### Data sources

The gene expression data from the GSE7476 and GSE13507 datasets and related clinical characteristics and prognostic information were downloaded from the Gene Expression Omnibus (GEO) database (https://www.ncbi.nlm.nih.gov/geo/). Differentially expressed genes were obtained through the “limma” package. CLIC3 was selected for further analysis. The expression data on CLIC3 mRNA was obtained from the GSE7476 and GSE13507 datasets. The clinicopathological and prognostic information of 165 BC patients was obtained from the GSE13507 dataset. The expression of CLIC3 mRNA in BC and normal tissues was verified using the Oncomine (https://www.oncomine.org) and The Cancer Genome Atlas (TCGA) database https://cancergenome.nih.gov/. Differentially expressed genes were obtained through the “DESeq” package from TCGA database. Immunohistochemical images were obtained from the Human Protein Atlas (HPA) database (https://www.proteinatlas.org/). The relationship between CLIC3 mRNA expression and overall survival in BC patients was analyzed by the Gene Expression Profiling Interactive Analysis (GEPIA) database (http://gepia.cancer-pku.cn/).

### Gene Ontology (GO) and Kyoto Encyclopedia of Genes and Genomes (KEGG) analyses of the genes coexpressed with CLIC3

To predict the possible functions and pathways of CLIC3 by studying the pathways of its coexpressed genes, the genes coexpressed with CLIC3 were retrieved and extracted from the Multi Experiment Matrix (MEM) database (http://biit.cs.ut.ee/mem/index.cgi) and cBioPortal (http://www.cbioportal.org/). The coexpressed genes were then taken together and displayed in a Venn diagram (http://bioinfogp.cnb.csic.es/tools/venny/). The coexpressed genes were entered into the Database for Annotation, Visualization and Integrated Discovery (DAVID, https://david.ncifcrf.gov/, Version 6.8) for GO and KEGG analyses. The critical value of significance for the gene enrichment and pathway screening was set at *P* < 0.05.

### GSEA

GSEA is a computational method used to determine whether a group of genes defined a priori show statistically significant and consistent differences between two biological states (Subramanian et al., 2005). GSEA was performed using GSEA3.0 (http://www.broad.mit.edu/gsea/) to investigate the effect of CLIC3 mRNA expression levels on biomarker gene sets in patients and to explore the possible link between CLIC3 and the pathways in BC. The BC dataset GSE7476 was divided into a high expression group and a low expression group according to the expression level of CLIC3 mRNA. The C2.cp.kegg.v6.2.symbols.gmt dataset was obtained from the Molecular Signatures Database (MsigDB) at the gene enrichment analysis website. Then, the enrichment analysis was performed according to the method of default weighted enrichment statistics, and the analysis was repeated 1,000 times at a time.

### Collection of human tissue specimens

In this study, between January 2019 and June 2019, eleven samples of BC tissues and adjacent normal tissues were collected from the Department of Urology, Affiliated Haikou Hospital of Xiangya Medical College, Central South University. These tissues were used to detect the expression level of CLIC3 mRNA by qRT-PCR. Verbal consent from all patients and approval from the ethics committee of Affiliated Haikou Hospital of Xiangya Medical College, Central South University were obtained before collection. The approval number is 2016(research ethical review)-005.

### qRT-PCR

Total RNA and cDNA were prepared with TRIzolReagent (Vazyme, Nanjing, China), TransScript One-Step gDNA Removal (Vazyme) and cDNA Synthesis SuperMix (Vazyme) according to the manufacturer’s instructions. cDNA was amplified on the LightCycler 480II system by using SYBR qPCR Master Mix (Vazyme). The oligonucleotide sequences of the primers used for qRT-PCR are as follows: CLIC3 (forward: 5′-CAGATCGAGGACTTTCTGGAG-3′, reverse: 5′-GGAGAACTTGTGGAAAACGTC-3′) and GAPDH (forward: 5′-CAGGAGGCATTGCTGATGAT-3′, reverse: 5′-GAAGGCTGGGGCTCATTT-3′). GAPDH was used as an internal control to standardize cycle threshold (CT) values to estimate gene expression. Relative mRNA expression was calculated using the 2^−ΔΔ*ct*^ method. Each sample was processed in triplicate to ensure quantitative accuracy.

### Statistical analysis

Statistical analyses were performed using SPSS (version 18.0), GraphPad Prism (version 7.0) and R software (version 3.5.1). T-tests were used to compare the expression of CLIC3 mRNA between BC tissues and normal bladder tissues. The relationship between CLIC3 and the clinicopathological characteristics of BC patients was analyzed using the chi-square test. The overall survival and cancer-specific survival of BC patients were analyzed using the log-rank test with the Kaplan–Meier method. Cox univariate and multivariate analyses of the overall survival and cancer-specific survival of BC patients were performed. *P* < 0.05 was considered statistically significant. A gene set of with *P* < 0.05 and a false discovery rate (FDR) <0.25 in the GSEA was judged to be a significantly enriched gene set.

## Results

### Expression level of CLIC3 mRNA in BC

**Figure 1:**
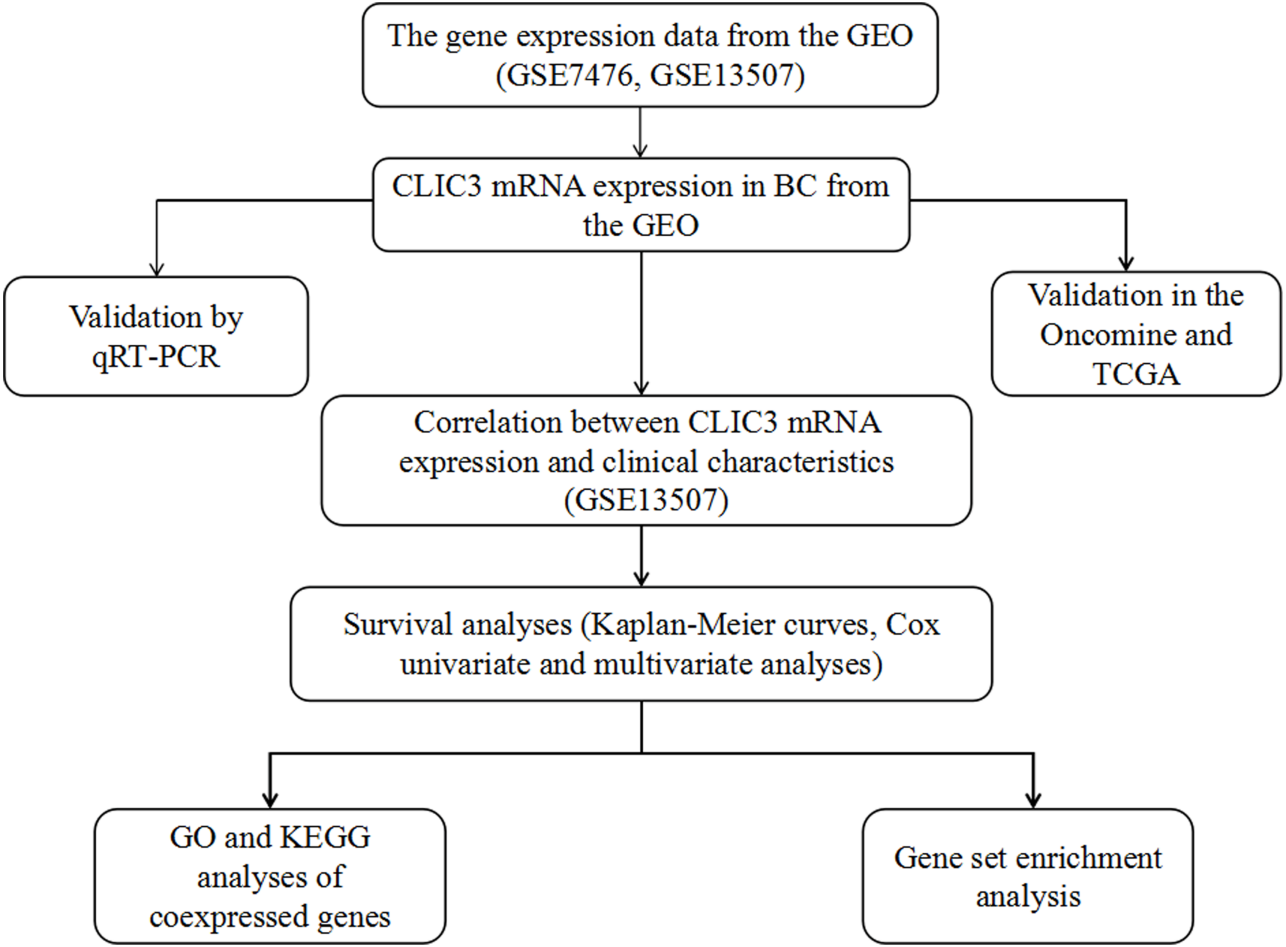
The flow chart of our research. CLIC3, chloride intracellular channel 3; GEO, Gene Expression Omnibus; TCGA, The Cancer Genome Atlas; BC, bladder cancer; qRT-PCR, quantitative real-time polymerase chain reaction; GO, Gene Ontology; KEGG, Kyoto Encyclopedia of Genes and Genomes.

**Figure 2:**
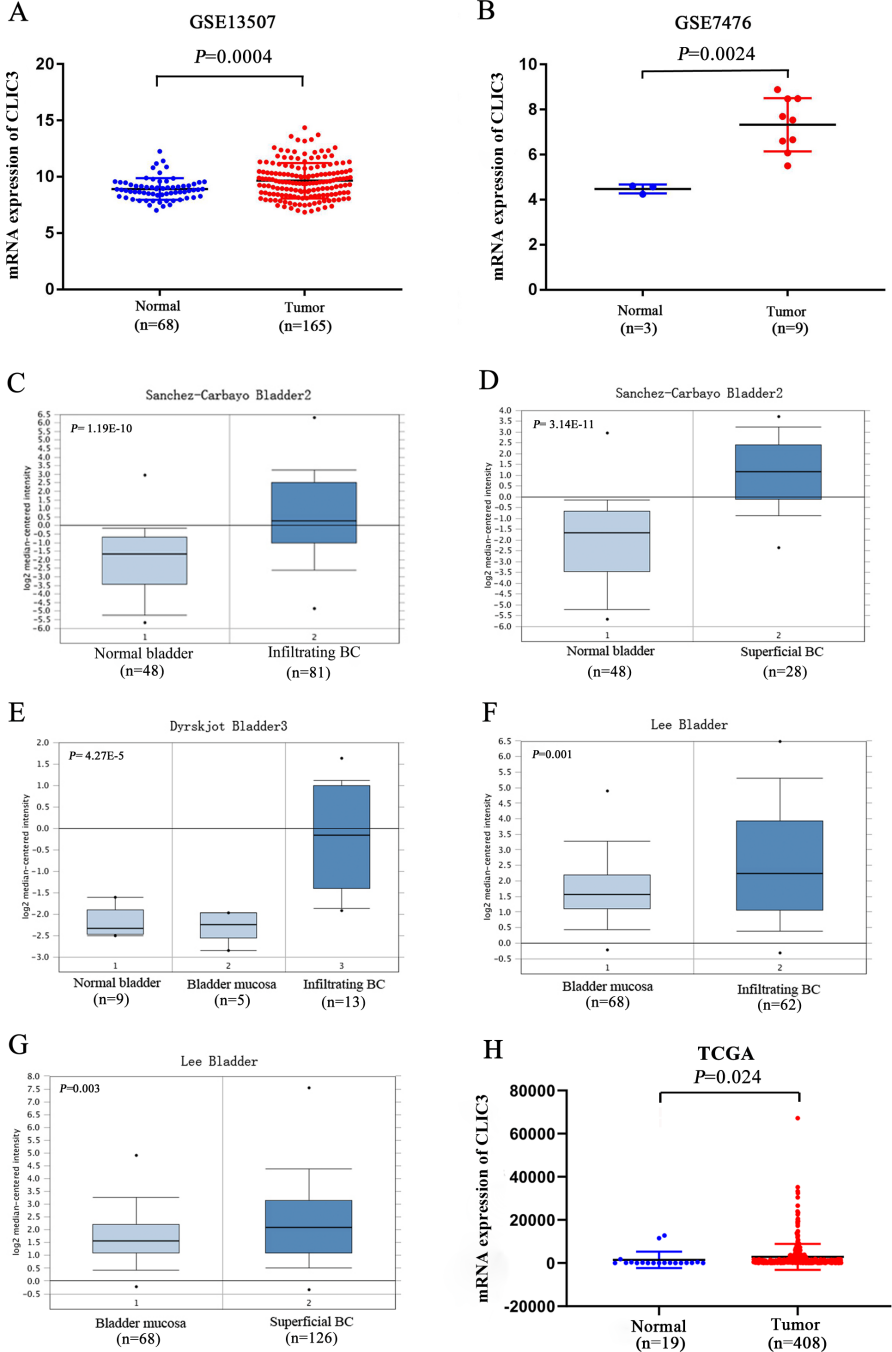
(A) Expression of CLIC3 mRNA in BC from the GSE13507 dataset. (B) Expression of CLIC3 mRNA in BC from the GSE7476 dataset. (C) Expression of CLIC3 in infiltrating BC from the ‘Sanchez-Carbayo Bladder 2’ dataset. (D) Expression of CLIC3 mRNA in superficial BC from the ‘Sanchez-Carbayo Bladder 2’ dataset. (E) Expression of CLIC3 mRNA in infiltrating BC from the ‘Dyrskjot Bladder 3’ dataset. (F) Expression of CLIC3 mRNA in infiltrating BC from the ‘Lee Bladder’ dataset. (G) Expression of CLIC3 mRNA in superficial BC from the ‘Lee Bladder’ dataset. (H) Expression of CLIC3 mRNA in BC from the TCGA. CLIC3, chloride intracellular channel 3; BC, bladder cancer; TCGA, The Cancer Genome Atlas.

Figure 1 shows the flow chart of our research. To explore the differential expression of CLIC3 mRNA in BC tissues and normal tissues, two GEO datasets (GSE13507 and GSE7476) were used. GSE13507 includes 165 tumor tissue samples and 68 normal tissue samples, and GSE7476 includes 9 tumor tissue samples and 3 normal tissue samples. The results showed that the expression of CLIC3 mRNA in BC tissues was significantly higher than that in normal tissues (Figs. 2A–2B, *P* < 0.01). In Oncomine, the ‘Sanchez-Carbayo Bladder 2’ dataset contains 48 normal tissue samples, 81 infiltrating BC samples and 28 superficial BC tissue samples. The ‘Dyrskjot Bladder 3’ dataset contains 9 normal tissue samples, 5 bladder mucosa samples and 13 infiltrating BC samples. The ‘Lee Bladder’ dataset contains 68 bladder mucosa samples, 62 infiltrating BC samples and 126 superficial BC tissue samples. Data from the Oncomine database indicated that CLIC3 mRNA expression was higher in BC tissue than in normal tissue (Figs. 2C–2G, *P* < 0.01). Data from the TCGA database also indicated that CLIC3 mRNA expression in BC tissues was higher than that in normal tissues (Fig. 2H, *P* = 0.024). The above studies indicated that the expression of CLIC3 mRNA in BC tissues was significantly higher than that in normal tissues. The immunohistochemical images from the HPA database showed that CLIC3 protein was expressed in the nuclear and cytoplasmic or cytomembrane and no all the tumors are positive for CLIC3 (Fig. S1).

### Correlation between CLIC3 mRNA expression and the clinical characteristics of BC patients

To study the correlation between CLIC3 mRNA expression and the clinical characteristics of BC patients, clinical data of 165 patients with BC were collected from the GSE13507 dataset. The results showed that CLIC3 mRNA expression was significantly correlated with age (*P* = 0.021, Table 1) and grade (*P* = 0.045, Table 1).

**Table 1:** Correlation between CLIC3 mRNA expression and clinicopathological variables in BC patients.

Clinical variables	CLIC3 mRNA expression		Chi-square	*P* value
	Low (%); *n* = 83	High (%); *n* = 82		
Age				
<65	42 (50.6)	27 (32.9)		
>=65	41 (49.4)	65 (67.1)	5.297	0.021
T stage				
T0∼T1	54 (65.1)	50 (60.9)		
T2∼T4	29 (34.9)	32 (39.1)	0.295	0.587
N stage				
N0	78 (94.0)			
N1∼N3	5 (6.0)	10 (12.2)	1.901	0.168
M stage				
M0	81 (97.6)	77 (93.9)		
M1	2 (2.4)	5 (6.1)	0.622	0.430
Grade				
Low	59 (71.1)	46 (56.1)		
High	24 (28.9)	36 (43.9)	4.004	0.045

**Notes.**

CLIC3 chloride intracellular channel 3 BC bladder cancer T stage stage of tumor invasion N stage stage of regional lymph node invasion M stage stage of metastasis

### Relationship between CLIC3 mRNA expression and the survival time of patients with BC

**Figure 3:**
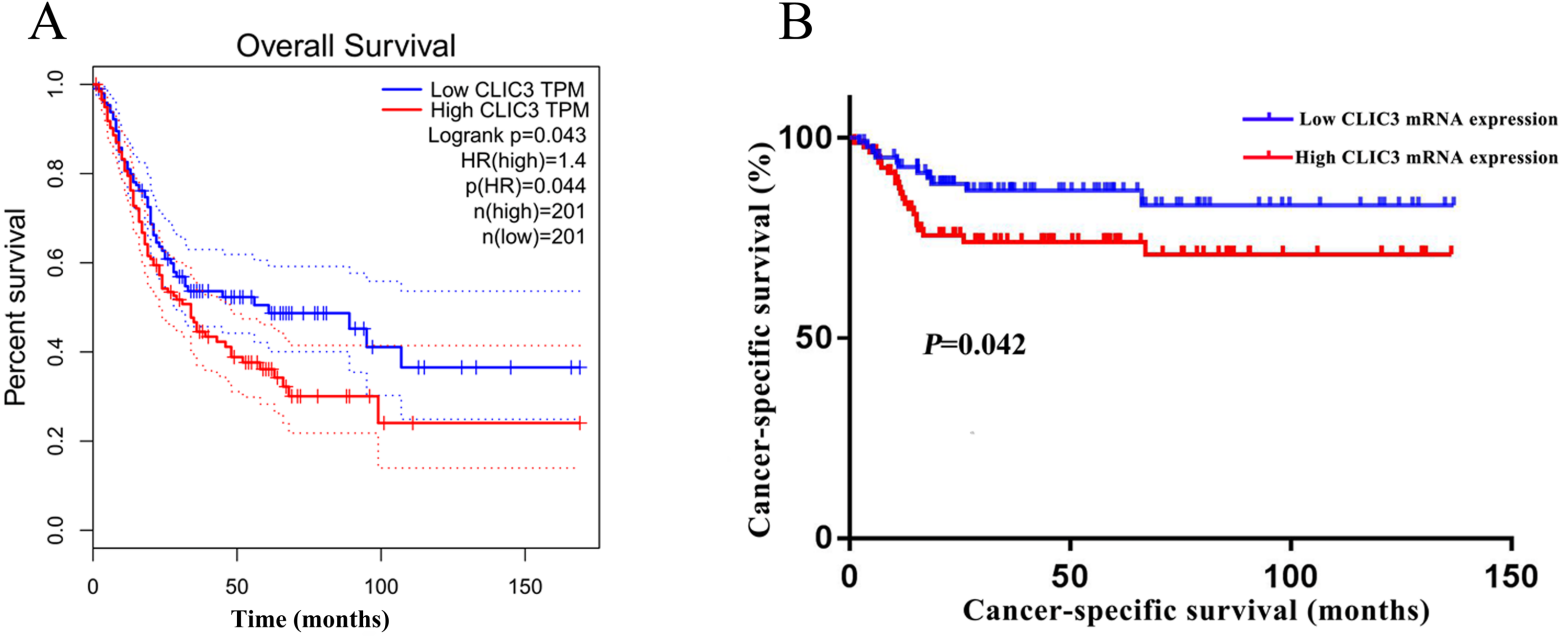
Relationship between CLIC3 mRNA expression and the prognosis of BC patients. (A) Relationship between CLIC3 mRNA expression and the overall survival of BC patients. (B) Relationship between CLIC3 mRNA expression and the cancer-specific survival of BC patients. CLIC3, chloride intracellular channel 3; BC, bladder cancer.

To elucidate the relationship between CLIC3 and prognosis in patients with BC, overall survival in BC patients was analyzed with the GEPIA database. The results showed that the overall survival time of patients with high CLIC3 mRNA expression was significantly shorter than that of patients with low CLIC3 mRNA expression (Fig. 3A, *P* = 0.044). The relationship between the expression of CLIC3 mRNA and tumor-specific survival in 165 BC patients was analyzed. The results showed that patients with high CLIC3 mRNA expression had shorter tumor-specific survival time (Fig. 3B, *P* = 0.042). These results indicated that CLIC3 mRNA expression may be prognostic factor for poor outcome in patients with BC.

### Cox univariate and multivariate analyses of overall survival and cancer-specific survival in BC patients

Cox univariate analysis showed that invasiveness, N stage and systemic chemo were associated with overall survival (Table 2); CLIC3 mRNA expression, gender and invasiveness were associated with tumor-specific survival (Table 3). The multivariate analysis showed that invasiveness, N stage, and M stage were associated with overall survival (Table 2); CLIC3 mRNA expression, gender, invasiveness, and N stage were associated with tumor-specific survival (Table 3). These results indicated that CLIC3 was an independent factor influencing the tumor-specific survival of BC patients.

**Table 2:** Cox univariate and multivariate analyses of the overall survival of 165 BC patients.

Clinical variables	Univariate analysis			Multivariate analysis		
	HR	95% CI	*P*	HR	95% CI	*P*
CLIC3 (low/high)	1.278	0.760–2.149	0.354	–	–	–
Gender (female/male)	0.609	0.337–1.098	0.099	–	–	–
Invasiveness (superficial/invasive)	2.453	1.323–4.550	0.004	2.374	1.285-4.388	0.006
T stage (T1–T4 vs T0)	2.324	0.869–6.214	0.093	2.377	0.911-6.204	0.077
N stage (N1–N3 vs N0)	2.771	1.014–7.569	0.047	3.065	1.170-8.026	0.023
M stage (M1 vs M0)	3.276	0.998–10.754	0.050	3.485	1.083-11.218	0.036
Grade (low/high)	1.046	0.622–1.761	0.865	–	–	–
Systemic chemo (no/yes)	0.461	0.214–0.996	0.049	0.484	0.228-1.027	0.059

**Notes.**

CLIC3 chloride intracellular channel 3 BC bladder cancer HR hazard ratio CI confidence interval T stage stage of tumor invasion N stage stage of regional lymph node invasion M stage stage of metastasis

**Table 3:** Cox univariate and multivariate analyses of the cancer-specific survival of 165 BC patients.

Clinical variables	Univariate analysis			Multivariate analysis		
	HR	95% CI	*P* value	HR	95% CI	*P* value
CLIC3 (low/high)	2.427	1.064–5.539	0.035	2.310	1.058–5.045	0.036
Gender (female/male)	0.416	0.187–0.929	0.032	0.448	0.206–0.976	0.043
Invasiveness(superficial/invasive)	24.282	5.940–99.268	0.000	18.333	5.292–63.516	0.001
T stage (T1–T4 vs T0)	0.710	0.072–6.982	0.769	–	–	–
N stage (N1–N3 vs N0)	2.571	0.919–7.188	0.072	3.138	1.398–7.044	0.006
M stage (M1 vs M0)	2.299	0.693–7.625	0.173	–	–	–
Grade (low/high)	1.037	0.486–2.213	0.924	–	–	–
Systemic chemo (no/yes)	0.570	0.242–1.344	0.199	–	–	–

**Notes.**

CLIC3 chloride intracellular channel 3 BC bladder cancer HR hazard ratio CI confidence interval T stage stage of tumor invasion N stage stage of regional lymph node invasion M stage stage of metastasis

### GO and KEGG analyses of the genes coexpressed with CLIC3

**Figure 4:**
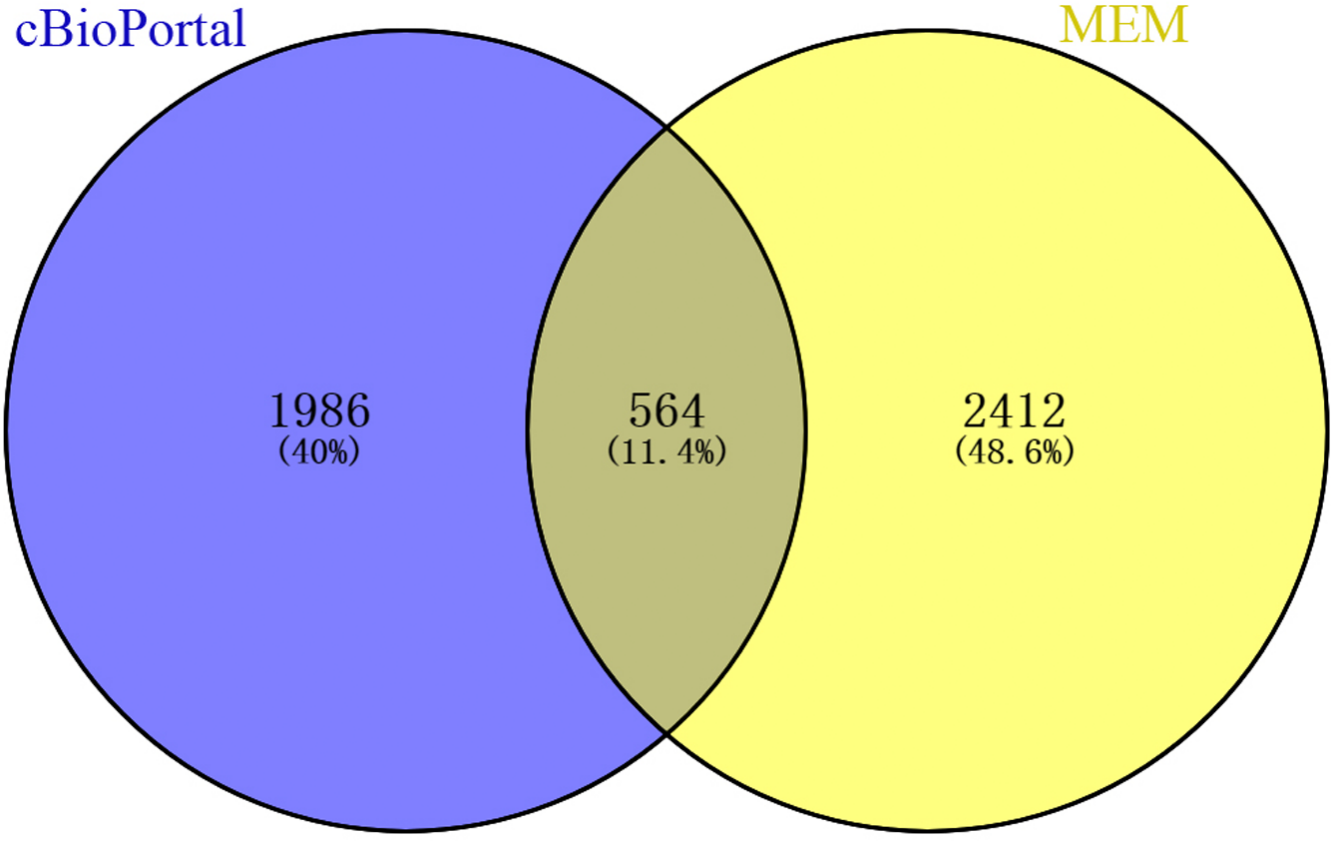
Venn diagram of coexpressed genes obtained from MEM and cBioPortal. MEM, Multi Experiment Matrix.

A total of 2,976 genes with at least two independent gene probes coexpressed with CLIC3 were extracted from the MEM database (*P* < 0.05). There were 2,550 genes with a Spearman correlation coefficient greater than 0.2 in cBioPortal (*P* < 0.0001). The coexpressed genes obtained from the above databases were intersected in the Venn diagram, and 564 genes were obtained for further analysis (Fig. 4).

**Figure 5:**
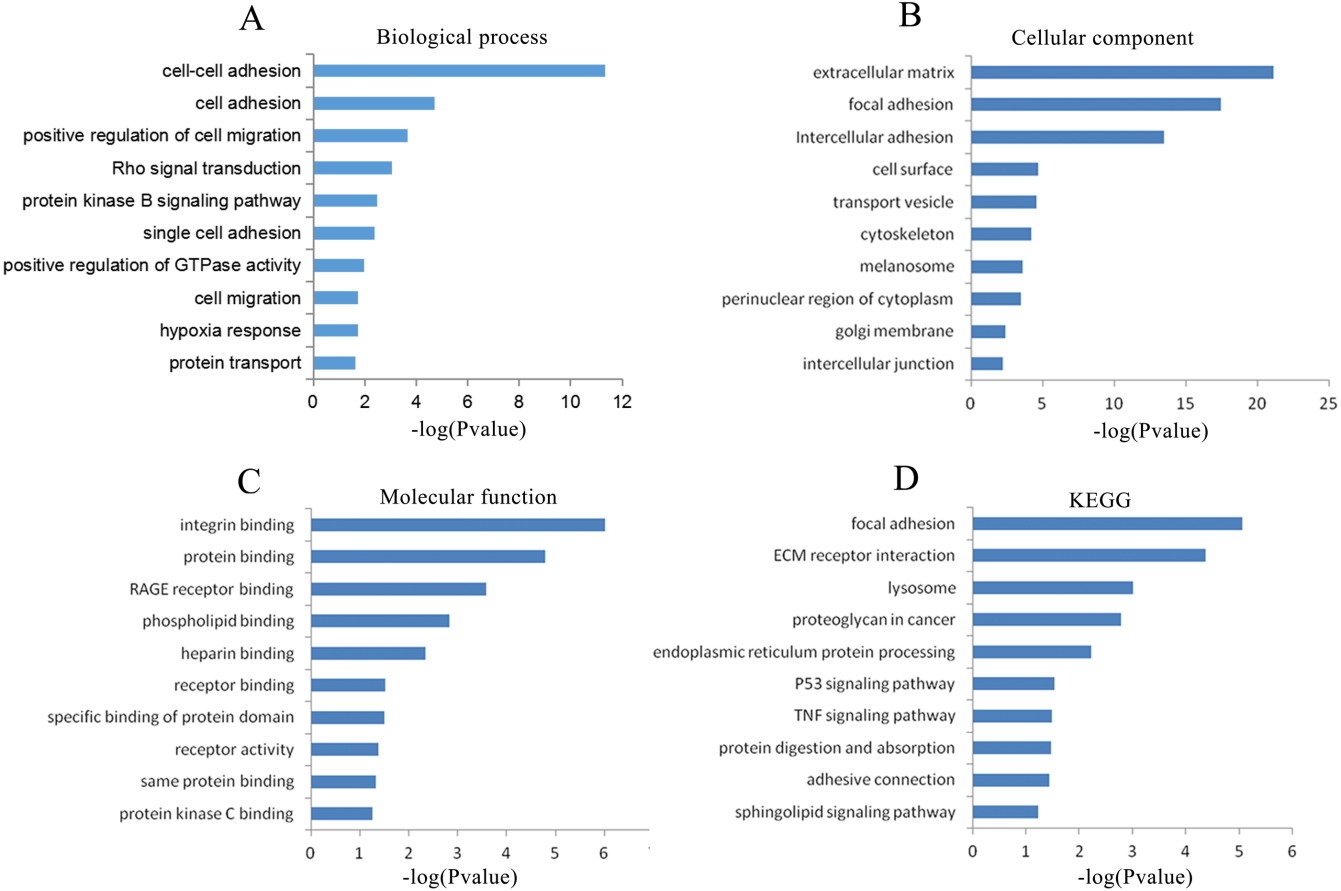
GO and KEGG analyses of the genes coexpressed with CLIC3. (A) Biological process. (B) Cellular component. (C) Molecular function. (D) KEGG pathway. CLIC3, chloride intracellular channel 3; GO, gene ontology; KEGG, Kyoto Encyclopedia of Genes and Genomes.

To explore the possible mechanisms in which CLIC3 may be involved in BC, a cluster analysis of the genes coexpressed with CLIC3 was performed to predict the function of CLIC3. A total of 564 coexpressed genes were analyzed by GO and KEGG. GO analysis revealed that these coexpressed genes mainly enriched in the biological processes of cell adhesion, the positive regulation of cell migration, Rho signaling transduction, the protein kinase B signaling pathway, cell migration and protein transport (Fig. 5A). The coexpressed genes mainly enriched in the cellular components of focal adhesion, intercellular adhesion and intercellular junction (Fig. 5B). The coexpressed genes mainly enriched in the molecular functions of integrin binding, protein binding and receptor binding (Fig. 5C). The coexpressed genes mainly enriched in focal adhesion, the extracellular matrix (ECM) receptor interaction, the P53 signaling pathway and the TNF signaling pathway according to KEGG analysis (Fig. 5D).

### Signal transduction pathways associated with CLIC3 mRNA expression

**Figure 6:**
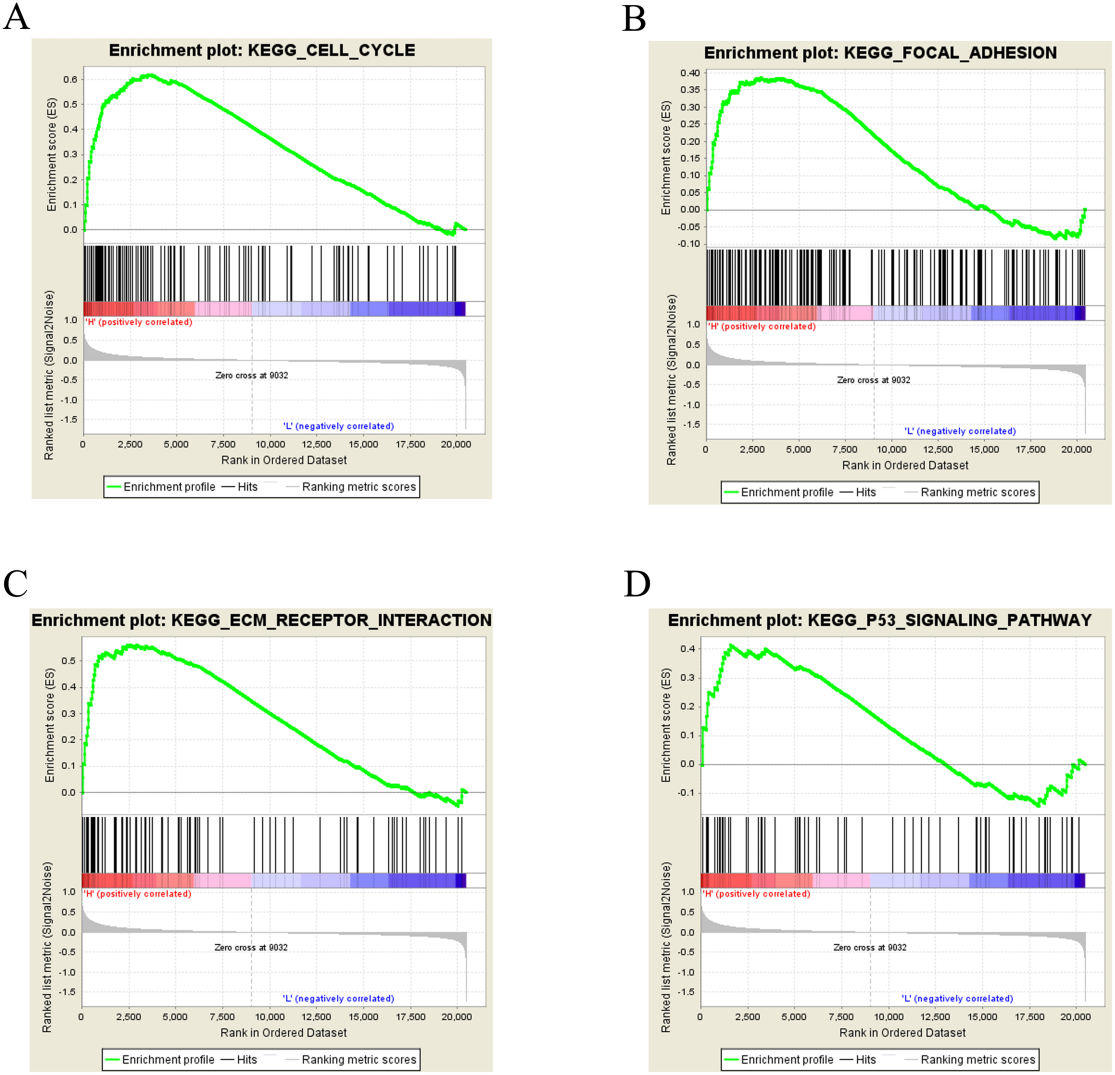
Enrichment plots from GSEA. (A) Cell cycle. (B) Focal adhesion. (C) ECM receptor interaction. (D) P53 signaling pathway. GSEA, gene set enrichment analysis; ECM, extracellular matrix; KEGG, Kyoto Encyclopedia of Genes and Genomes.

To further study CLIC3-associated signaling pathways in BC, GSEA was performed. The results showed that the cell cycle, focal adhesion, the ECM receptor interaction and the P53 signaling pathway were significantly enriched in the high CLIC3 mRNA expression phenotype (Figs. 6A–6D, Table 4). These results suggested that CLIC3 may be significantly associated with cell cycle, focal adhesion, the ECM receptor interaction and the P53 signaling pathway. The GSEA results provide a good basis for understanding how CLIC3 plays a role in BC.

**Table 4:** High CLIC3 mRNA expression phenotype enriched gene set.

Gene set	ES	NES	NOM *p*-val	FDR *q*-val
Cell cycle	0.619	2.247	0.000	0.000
Focal adhesion	0.387	1.482	0.003	0.188
ECM receptor interaction	0.561	1.899	0.000	0.004
P53 signaling pathway	0.415	1.370	0.045	0.215

**Notes.**

CLIC3 chloride intracellular channel 3 ES enrichment score NES normalized enrichment score

### Validation of CLIC3 mRNA expression in BC by qRT-PCR

**Figure 7:**
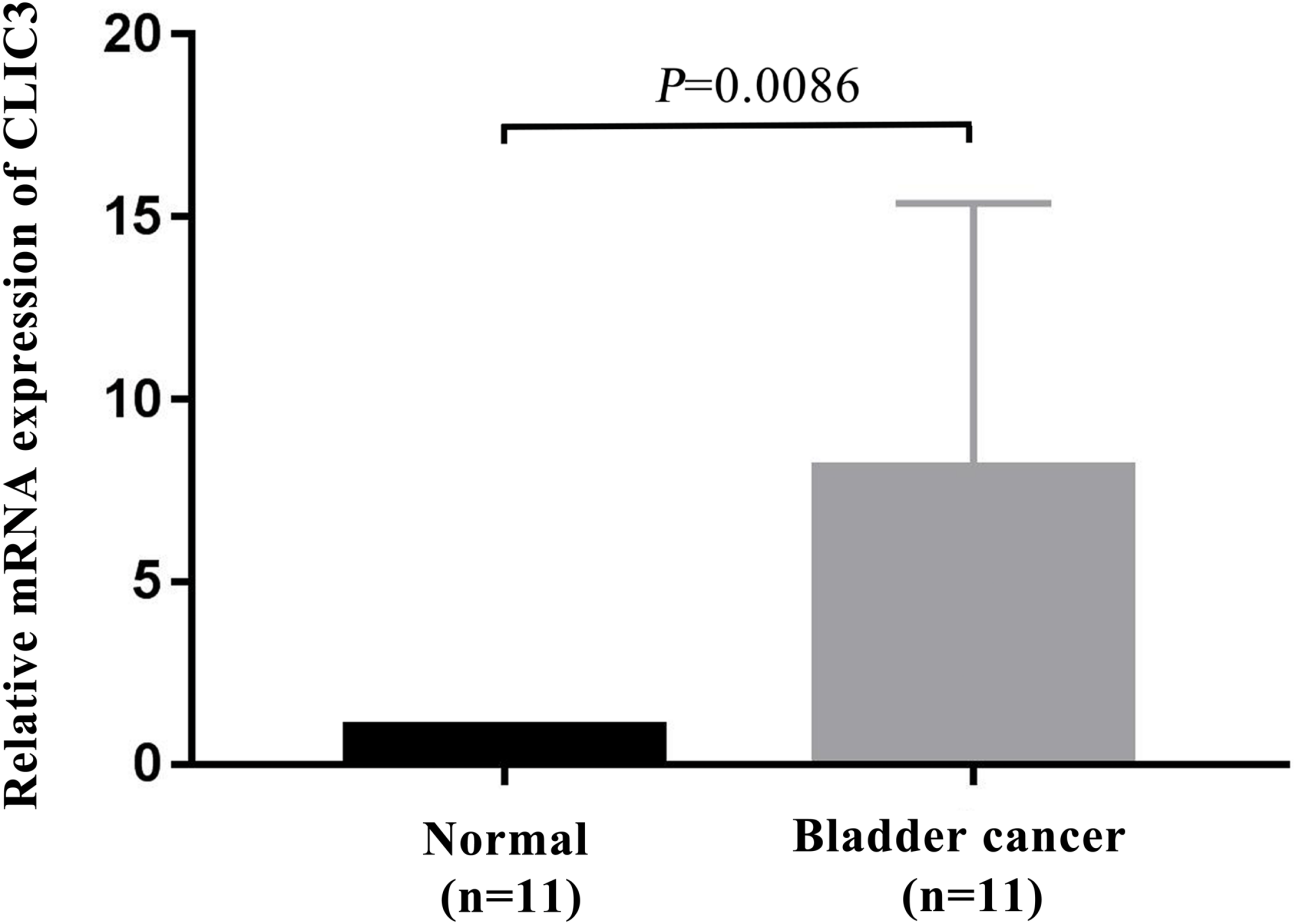
CLIC3 mRNA expression was analyzed in 11 paired tumor and normal tissues by qRT-PCR. CLIC3, chloride intracellular channel 3; qRT-PCR, quantitative real-time polymerase chain reaction.

To further verify the expression of CLIC3 mRNA in BC, 11 pairs of BC tissues and adjacent normal tissues were selected for detection by qRT-PCR. The results showed that the expression level of CLIC3 mRNA in BC tissues was significantly higher than that in normal tissues (Fig. 7, *P* = 0.0086).

## Discussion

BC is one of the most common malignant tumors in the genitourinary system, most of which originates from the urothelium, and the morbidity and mortality of men are higher than those of women (Kamat et al., 2016). Non-muscle invasive BC is the most common pathological tissue type, and 20% to 25% of patients have poor prognosis and can be further developed into MIBC (Chavan et al., 2014). The malignant degree and metastasis rate of MIBC are very high (Kamat et al., 2016). Due to the lack of specific molecular targets for BC, it is urgent to find some genes with different expression and clinical significance to explore the occurrence and development of BC and to provide theoretical basis for further studies on new therapeutic targets.

CLIC3 is a member of the members of the intracellular chloride channel family that stimulates chloride conductance and cell growth (Qian et al., 1999). CLIC3 is highly expressed in various tumors and promotes tumor development, leading to the poor prognosis of patients. Tasiopoulou et al. (2015) used the gene expression data from a microarray dataset to compare the differential expression profiles of CLIC1-6 between malignant pleural mesothelioma and normal tissues. The results showed that the gene expression of CLIC3 and CLIC4 was significantly increased in malignant pleural mesothelioma tissues compared with normal tissues. Studies have shown that CLIC3 determines breast cancer invasion and metastasis by controlling late endosomal matrix metalloproteinase MT1-MMP. High CLIC3 expression predicts a poor prognosis of estrogen receptor-negative breast cancer (Macpherson et al., 2014). CLIC3 and Rab25 synergistically promote late endosomal/lysosomal integrin cycling and drive cancer progression (Dozynkiewicz et al., 2012). In addition, CLIC3 is highly expressed in mucoepidermoid carcinoma of the salivary gland and promotes tumor progression (Wang et al., 2015). Our study found that CLIC3 is closely linked to BC and analyzed its possibility as a prognostic marker.

In our study, we used the GSE13507 and GSE7476 datasets from the GEO database and found that CLIC3 mRNA is highly expressed in BC tissues. These findings were then validated in other databases and qRT-PCR. By extracting expression data of CLIC3 mRNA in BC and normal tissues from the Oncomine database, studies by Sanchez-Carbayo et al. (2006), Dyrskjot et al. (2004) and Lee et al. (2010) showed that the expression of CLIC3 mRNA in BC was significantly higher than that in normal tissues. TCGA and qRT-PCR results also showed that the expression of CLIC3 mRNA in BC tissues was higher than that in normal bladder tissues. By analyzing the clinicopathological data from the GSE13507 dataset, we found that CLIC3 mRNA expression was correlated with grade and age. Further analysis revealed that patients with high CLIC3 mRNA expression had lower overall survival and tumor-specific survival. Cox univariate and multivariate analyses showed that CLIC3 was an independent factor influencing the tumor-specific survival of BC patients. Deng et al. (2018) attempted to predict the potential mechanism of HOXA13 action in lung adenocarcinoma by performing GO and KEGG analyses on HOXA13 co-expressed genes. To study the possible role of CLIC3 in BC, 564 coexpressed genes obtained from the intersection of cBioPortal and MEM databases were imported into the DAVID online tool to predict the pathway these genes are involved in. KEGG analysis showed that the coexpressed genes were significantly enriched in focal adhesion, the ECM receptor interaction, and the P53 signaling pathway. GSEA is able to obtain biological pathways for gene involvement (Mootha et al., 2003). GSEA also indicated that the cell cycle, focal adhesion, the ECM receptor interaction and the P53 signaling pathway were significantly enriched in the high CLIC3 expression phenotype. CLIC3 may not be responsible of the changes and may be a consequence of deregulated signaling pathways driving BC. These results provide a good experimental basis for the role of CLIC3 in BC. According to the literature, the focal adhesion pathway regulates cell migration and metastasis (Eke & Cordes, 2015). The P53 signaling pathway plays an important role in the proliferation and apoptosis of BC (Zhang et al., 2018). This study also has some limitations (e.g., a lack basic research on relevant biological mechanisms and only RNA expression data), and more experiments are needed to further verify these findings in the future.

## Conclusions

CLIC3 mRNA is highly expressed in BC and is a marker of a poor prognosis in BC patients. CLIC3 may be significantly associated with the cell cycle, focal adhesion, the ECM receptor interaction and the P53 signaling pathway in BC. However, the specific mechanism of action of CLIC3 in the development of BC must be verified in further experiments.

##  Supplemental Information

10.7717/peerj.8348/supp-1 Figure S1 IHC analysis of CLIC3 protein expression in BC in the HPA database (A) Negative expression of CLIC3 protein was observed in BC, in which no tumor cells demonstrated staning of CLIC3. (B) Low expression of CLIC3 protein was observed in BC, in which <20% of tumor cells demonstrated staining of CLIC3. (C) Medium expression of CLIC3 protein was observed in BC. IHC: immunohistochemical; HPA: Human Protein Atlas; CLIC3: chloride intracellular channel 3; BC, bladder cancer.

10.7717/peerj.8348/supp-2 Data S1 Raw data

## Additional Information and Declarations

### Data Availability

The following information was supplied regarding data availability: Raw data is available in the Supplemental Files.

## References

[ref-1] Chavan S, Bray F, Lortet-Tieulent J, Goodman M, Jemal A (2014). International variations in bladder cancer incidence and mortality. European Urology.

[ref-2] Deng Y, He R, Zhang R, Gan B, Zhang Y, Chen G, Hu X (2018). The expression of HOXA13 in lung adenocarcinoma and its clinical significance: a study based on The Cancer Genome Atlas, Oncomine and reverse transcription-quantitative polymerase chain reaction. Oncology Letters.

[ref-3] Dozynkiewicz MA, Jamieson NB, Macpherson I, Grindlay J, Van den Berghe PV, Von Thun A, Morton JP, Gourley C, Timpson P, Nixon C, McKay CJ, Carter R, Strachan D, Anderson K, Sansom OJ, Caswell PT, Norman JC (2012). Rab25 and CLIC3 collaborate to promote integrin recycling from late endosomes/lysosomes and drive cancer progression. Developmental Cell.

[ref-4] Dyrskjot L, Kruhoffer M, Thykjaer T, Marcussen N, Jensen JL, Moller K, Orntoft TF (2004). Gene expression in the urinary bladder: a common carcinoma *in situ* gene expression signature exists disregarding histopathological classification. Cancer Research.

[ref-5] Eke I, Cordes N (2015). Focal adhesion signaling and therapy resistance in cancer. Seminars in Cancer Biology.

[ref-6] Fernandez-Salas E, Sagar M, Cheng C, Yuspa SH, Weinberg WC (1999). p53 and tumor necrosis factor alpha regulate the expression of a mitochondrial chloride channel protein. Journal of Biological Chemistry.

[ref-7] Hernandez-Fernaud JR, Ruengeler E, Casazza A, Neilson LJ, Pulleine E, Santi A, Ismail S, Lilla S, Dhayade S, MacPherson IR, McNeish I, Ennis D, Ali H, Kugeratski FG, Al Khamici H, Van den Biggelaar M, Van den Berghe PV, Cloix C, McDonald L, Millan D, Hoyle A, Kuchnio A, Carmeliet P, Valenzuela SM, Blyth K, Yin H, Mazzone M, Norman JC, Zanivan S (2017). Secreted CLIC3 drives cancer progression through its glutathione-dependent oxidoreductase activity. Nature Communications.

[ref-8] Kamat AM, Hahn NM, Efstathiou JA, Lerner SP, Malmstrom PU, Choi W, Guo CC, Lotan Y, Kassouf W (2016). Bladder cancer. Lancet.

[ref-9] Kim WJ (2015). Is 5 -AMP-activated protein kinase both Jekyll and Hyde in bladder cancer?. International Neurourology Journal.

[ref-10] Kiyoshima K, Akitake M, Shiota M, Takeuchi A, Takahashi R, Inokuchi J, Tatsugami K, Yokomizo A, Eto M (2016). Prognostic significance of preoperative urine cytology in low-grade non-muscle-invasive bladder cancer. Anticancer Research.

[ref-11] Kunzelmann K (2005). Ion channels and cancer. Journal of Membrane Biology.

[ref-12] Lee JS, Leem SH, Lee SY, Kim SC, Park ES, Kim SB, Kim SK, Kim YJ, Kim WJ, Chu IS (2010). Expression signature of E2F1 and its associated genes predict superficial to invasive progression of bladder tumors. Journal of Clinical Oncology.

[ref-13] Macpherson IR, Rainero E, Mitchell LE, Van den Berghe PV, Speirs C, Dozynkiewicz MA, Chaudhary S, Kalna G, Edwards J, Timpson P, Norman JC (2014). CLIC3 controls recycling of late endosomal MT1-MMP and dictates invasion and metastasis in breast cancer. Journal of Cell Science.

[ref-14] Money TT, King RG, Wong MH, Stevenson JL, Kalionis B, Erwich JJ, Huisman MA, Timmer A, Hiden U, Desoye G, Gude NM (2007). Expression and cellular localisation of chloride intracellular channel 3 in human placenta and fetal membranes. Placenta.

[ref-15] Mootha VK, Lindgren CM, Eriksson KF, Subramanian A, Sihag S, Lehar J, Puigserver P, Carlsson E, Ridderstrale M, Laurila E, Houstis N, Daly MJ, Patterson N, Mesirov JP, Golub TR, Tamayo P, Spiegelman B, Lander ES, Hirschhorn JN, Altshuler D, Groop LC (2003). PGC-1alpha-responsive genes involved in oxidative phosphorylation are coordinately downregulated in human diabetes. Nature Genetics.

[ref-16] Murthi P, Stevenson JL, Money TT, Borg AJ, Brennecke SP, Gude NM (2012). Placental CLIC3 is increased in fetal growth restriction and pre-eclampsia affected human pregnancies. Placenta.

[ref-17] Qian Z, Okuhara D, Abe MK, Rosner MR (1999). Molecular cloning and characterization of a mitogen-activated protein kinase-associated intracellular chloride channel. Journal of Biological Chemistry.

[ref-18] Sanchez-Carbayo M, Socci ND, Lozano J, Saint F, Cordon-Cardo C (2006). Defining molecular profiles of poor outcome in patients with invasive bladder cancer using oligonucleotide microarrays. Journal of Clinical Oncology.

[ref-19] Siegel RL, Miller KD, Jemal A (2017). Cancer statistics. A Cancer Journal for Clinicians.

[ref-20] Subramanian A, Tamayo P, Mootha VK, Mukherjee S, Ebert BL, Gillette MA, Paulovich A, Pomeroy SL, Golub TR, Lander ES, Mesirov JP (2005). Gene set enrichment analysis: a knowledge-based approach for interpreting genome-wide expression profiles. Proceedings of the National Academy of Sciences of the United States of America.

[ref-21] Suh KS, Yuspa SH (2005). Intracellular chloride channels: critical mediators of cell viability and potential targets for cancer therapy. Current Pharmaceutical Design.

[ref-22] Tasiopoulou V, Magouliotis D, Solenov EI, Vavougios G, Molyvdas PA, Gourgoulianis KI, Hatzoglou C, Zarogiannis SG (2015). Transcriptional over-expression of chloride intracellular channels 3 and 4 in malignant pleural mesothelioma. Computational Biology and Chemistry.

[ref-23] Ulmasov B, Bruno J, Gordon N, Hartnett ME, Edwards JC (2009). Chloride intracellular channel protein-4 functions in angiogenesis by supporting acidification of vacuoles along the intracellular tubulogenic pathway. American Journal of Pathology.

[ref-24] Wang Z, Ling S, Rettig E, Sobel R, Tan M, Fertig EJ, Considine M, El-Naggar AK, Brait M, Fakhry C, Ha PK (2015). Epigenetic screening of salivary gland mucoepidermoid carcinoma identifies hypomethylation of CLIC3 as a common alteration. Oral Oncology.

[ref-25] Xu Y, Kang J, Yuan Z, Li H, Su J, Li Y, Kong X, Zhang H, Wang W, Sun L (2013). Suppression of CLIC4/mtCLIC enhances hydrogen peroxide-induced apoptosis in C6 glioma cells. Oncology Reports.

[ref-26] Zhang ZF, Zhang HR, Zhang QY, Lai SY, Feng YZ, Zhou Y, Zheng SR, Shi R, Zhou JY (2018). High expression of TMEM40 is associated with the malignant behavior and tumorigenesis in bladder cancer. Journal of Translational Medicine.

