# Supplementary figures and images for: Prognostic value of CLIC3 mRNA overexpression in bladder cancer
Source: PeerJ. 2020 Jan 6;8:e8348. doi: 10.7717/peerj.8348 (PMC6951294; doi:10.7717/peerj.8348)

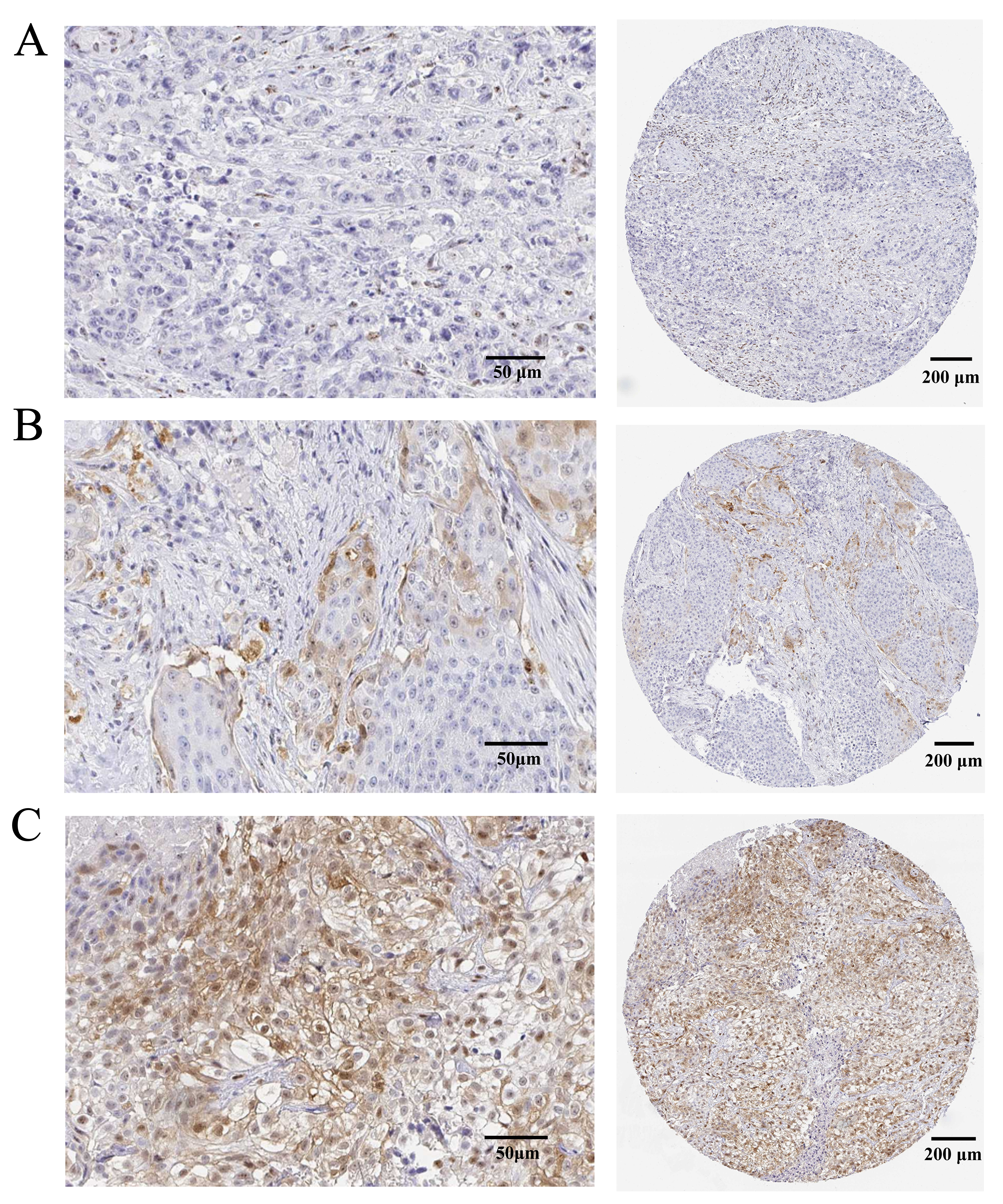

Supplement: Figure S1 — (A) Negative expression of CLIC3 protein was observed in BC, in which no tumor cells demonstrated staning of CLIC3. (B) Low expression of CLIC3 protein was observed in BC, in which <20% of tumor cells demonstrated staining of CLIC3. (C) Medium expression of CLIC3 protein was observed in BC. IHC: immunohistochemical; HPA: Human Protein Atlas; CLIC3: chloride intracellular channel 3; BC, bladder cancer. [file peerj-08-8348-s001.png]
